# Early recanalization in large-vessel occlusion stroke patients transferred for endovascular treatment

**DOI:** 10.1136/neurintsurg-2021-017441

**Published:** 2021-05-13

**Authors:** Nerea Arrarte Terreros, Agnetha A E Bruggeman, Isabella S J Swijnenburg, Laura C C van Meenen, Adrien E Groot, Jonathan M Coutinho, Yvo B W E M Roos, Bart J Emmer, Ludo F M Beenen, Ed van Bavel, Henk A Marquering, Charles B L M Majoie

**Affiliations:** 1 Department of Biomedical Engineering and Physics, Amsterdam UMC, location AMC, Amsterdam, the Netherlands; 2 Department of Radiology and Nuclear Medicine, Amsterdam UMC, location AMC, Amsterdam, the Netherlands; 3 Department of Neurology, Amsterdam UMC, location AMC, Amsterdam, the Netherlands

**Keywords:** stroke, thrombolysis, thrombectomy, CT, CT angiography

## Abstract

**Background:**

We performed an exploratory analysis to identify patient and thrombus characteristics associated with early recanalization in large-vessel occlusion (LVO) stroke patients transferred for endovascular treatment (EVT) from a primary (PSC) to a comprehensive stroke center (CSC).

**Methods:**

We included patients with an LVO stroke of the anterior circulation who were transferred to our hospital for EVT and underwent repeated imaging between January 2016 and June 2019. We compared patient characteristics, workflow time metrics, functional outcome (modified Rankin Scale at 90 days), and baseline thrombus imaging characteristics, which included: occlusion location, thrombus length, attenuation, perviousness, distance from terminus of intracranial carotid artery to the thrombus (DT), and clot burden score (CBS), between early-recanalized LVO (ER-LVO), and non-early-recanalized LVO (NER-LVO) patients.

**Results:**

One hundred and forty-nine patients were included in the analysis. Early recanalization occurred in 32% of patients. ER-LVO patients less often had a medical history of hypertension (31% vs 49%, P=0.04), and more often had clinical improvement between PSC and CSC (ΔNIHSS −5 vs 3, P<0.01), compared with NER-LVO patients. Thrombolysis administration was similar in both groups (88% vs 78%, P=0.18). ER-LVO patients had no ICA occlusions (0% vs 27%, P<0.01), more often an M2 occlusion (35% vs 17%, P=0.01), longer DT (27 mm vs 12 mm, P<0.01), shorter thrombi (17 mm vs 27 mm, P<0.01), and higher CBS (8 vs 6, P<0.01) at baseline imaging. ER-LVO patients had lower mRS scores (1 vs 3, P=0.02).

**Conclusions:**

Early recanalization is associated with clinical improvement between PSC and CSC admission, more distal occlusions and shorter thrombi at baseline imaging, and better functional outcome.

## Introduction

The imaging protocol to confirm the presence of an acute ischemic stroke (AIS) due to an intracranial large-vessel occlusion (LVO) generally includes non-contrast CT (NCCT) and CT angiography (CTA).^1^ If an LVO is detected at a primary stroke center (PSC), patients receive intravenous treatment with alteplase (IVT), if eligible, and are subsequently transferred to a comprehensive stroke center (CSC) for endovascular treatment (EVT).^1^ In part of these patients, imaging is repeated in the CSC, often because of clinical improvement or deterioration, or to obtain additional imaging characteristics.^2 3^ It has been observed that in some of the transferred patients, by the time the imaging protocol is repeated at the CSC, the thrombus has resolved, and therefore EVT is no longer indicated.^2 4–6^ Identifying patient characteristics associated with this early recanalization may improve treatment workflow of transferred patients. As an example, patients without a predisposition to early recanalization might benefit from direct treatment with EVT, avoiding the administration of IVT, and thus, the associated hemorrhagic complications, delay to EVT, and thrombus fragmentation during EVT.^7 8^ In addition, identifying early recanalization might reduce futile preparation for EVT procedures and the related invasive angiography imaging, procedural risks (such as arterial dissections), and costs.^9^ The goal of our study is to identify patient and thrombus characteristics that are associated with early recanalization. Therefore, we compare patient characteristics and quantify thrombus characteristics on pre-IVT CT imaging data of early recanalized LVO (ER-LVO) and non-early recanalized LVO (NER-LVO) patients. In addition, we examine differences in workflow-related time metrics and patient outcome between ER-LVO and NER-LVO patients.

## Methods

### Patient selection

We included adult patients with an LVO stroke of the anterior circulation who were presented to a PSC between January 2016 and June 2019, were subsequently transferred to our hospital (Amsterdam University Medical Centers, location AMC) for EVT, and underwent repeated neuroimaging on arrival.

This study was evaluated by the medical ethics review committee of our hospital, who waived the need for obtaining written informed consent. The procedures followed were all in accordance with institutional guidelines. A letter with detailed information about the study was sent to all patients meeting the inclusion criteria. The patient or legal representative had the opportunity to deny consent for use of their data via an opt-out form, conforming to the European Union General Data Protection Regulation.

### Definitions

An LVO was defined as the occlusion of one of the major intracranial arteries. Early recanalization was defined as the dissolution of the thrombus, as confirmed with neuroimaging performed at the CSC. Non-early recanalization was defined as a persistent LVO either in the same vessel segment (with or without distal migration) or in a new vascular territory. Spontaneous early recanalization was defined as early recanalization without IVT administration.

### Patient characteristics

Stroke severity at PSC and CSC admission was assessed using the National Institutes of Health Stroke Scale (NIHSS_PSC_ and NIHSS_CSC_, respectively). The change in NIHSS score between presentation to the PSC and presentation to the CSC was also calculated (ΔNIHSS=NIHSS_CSC_ – NIHSS_PSC_). A negative ΔNIHSS value means clinical improvement and a positive value implies clinical deterioration.

### Thrombus imaging characteristics

We assessed the occlusion location, the distance from terminus of intracranial carotid artery (ICA-T) to the thrombus (DT), thrombus length, thrombus attenuation, thrombus perviousness, and clot burden score (CBS). These characteristics were assessed on pre-IVT NCCT and/or CTA data from the PSC. If both NCCT and CTA were available, the scans were co-registered using Elastix rigid-registration.^10^ Scan co-registration permits to simultaneously measure the thrombus characteristics on both NCCT and CTA scans. In gantry-tilted scans, Elastix affine-registration was applied.^11^ For better scan co-registration, the scans were re-sliced to obtain a 2.5 mm slice thickness if the slice thickness was larger than 2.5 mm. Poor quality scans were defined as scans with movement artefacts, incomplete field of view, severe beam hardening artefacts, and/or metal artefacts.

Thrombus location was re-assessed on CTA by a trained observer cognizant of the radiology report of the attending neuroradiologist, supported by the hyperdense artery sign if observed on NCCT, and was classified as internal carotid artery (ICA), middle cerebral artery (M1, M2), or anterior cerebral artery (A1, A2) occlusions.

**Figure 1:**
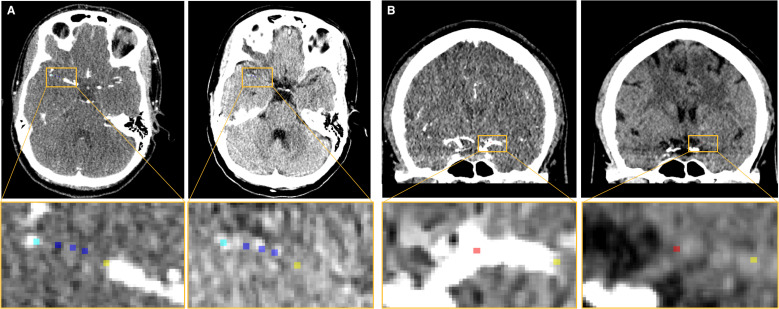
(A) CTA and NCCT scans of an AIS patient with a right MCA occlusion. Yellow and cyan markers indicate the proximal and distal border of the thrombus, respectively. The aim of the placement of the blue markers within the thrombus is twofold: aid in the computation of the thrombus length; and serve to create the spherical regions of interest for the thrombus attenuation and perviousness. (B) CTA and NCCT scans of an AIS patient with a left MCA occlusion. red and yellow markers indicate the location of the ICA-T and proximal thrombus border, respectively. AIS, acute ischemic stroke; CTA, CT angiography; ICA-T, terminus of intracranial carotid artery; MCA, middle cerebral artery; NCCT, non-contrast CT.

The DT was defined as the path length from the ICA-T to the most proximal thrombus border. Up to five markers were placed along the vessel centerline connecting the ICA-T to the proximal thrombus border using the software ITK-snap (version 3.4)^12^ (figure 1B). The distance was obtained by summation of the distances between adjacent markers. If the thrombus proximal border was located more proximal than the ICA-T, the DT was set to zero.

Thrombus length was defined as the largest extension of lack of contrast filling shown on CTA, aided by the hyperdense artery sign if observed on NCCT. We placed five markers along the occluded vessel centerline using ITK-snap,^12^ ensuring that the first and last markers were located at the most proximal and distal borders of the thrombus, respectively (figure 1A). The length was quantified by computing the path length between adjacent markers. In the case of bifurcating thrombi, the occluded branch with the longest thrombus was followed.

Thrombus perviousness is a measure of the amount of contrast that has penetrated the thrombus in CTA, compared with NCCT. Both thrombus attenuation and perviousness were measured by manually placing three spherical regions of interests of 1 mm radius within the thrombus (figure 1A). Thrombus attenuation was computed as the mean attenuation values of the regions of interests in NCCT. Thrombus perviousness was calculated by subtracting the mean attenuation values of the regions of interest on NCCT from CTA.^13^ For these computations, scans with severe re-slicing (eg, scans with a 5 mm thickness) and slight beam hardening artefacts were excluded since they may affect the measured Hounsfield Units (HU).

The CBS is a 10-point score that assesses the thrombus extension in the anterior circulation. The CBS was assessed as reported previously.^14^

All measurements were performed using both co-registered scans simultaneously. If only NCCT was available, DT, thrombus length, thrombus perviousness, and CBS were measured solely based on the hyperdense artery sign (if present). If only CTA was available, DT, thrombus length, and CBS were measured based on the lack of contrast filling. All measurements were performed by three trained observers: NAT, AAEB, and ISJS.

### Workflow time metrics

Workflow-related outcomes included time from stroke onset to initiation of IVT, time from stroke onset to arrival at the PSC, time from arrival at the PSC to initiation of IVT, and time from imaging at the PSC to imaging at the CSC. The time of stroke onset was defined as the time of witnessed symptom onset or, if unknown, as the time the patient was last seen well.

### Outcomes

Functional patient outcome was assessed using the modified Rankin Scale (mRS) at 90 days after stroke onset. Functional independence was defined as an mRS of 0–2.

### Statistical analysis

We performed an exploratory analysis and compared patient and thrombus characteristics of ER-LVO patients to those of NER-LVO patients. Data are presented as median and interquartile range (IQR) for continuous variables, and number and proportion (%) for categorical variables. Comparison between groups was done using the Mann–Whitney *U* test for continuous variables and χ^2^ and Fisher’s exact test for categorical variables. Statistical significance was set at P<0.05. All the analyses were performed with IBM SPPS Statistics package software (version 26.0).

## Results

### Patient characteristics

In total, 677 patients were transferred to our hospital for EVT between January 2016 and June 2019. Of these, 169/667 (25%) underwent repeated CT imaging on arrival. The reasons underlying repeated imaging in our study population have previously been reported:^3^ clinical improvement (52%), clinical deterioration (40%), and other reasons (8%). We excluded 20 patients due to: objection to use of data (4/169), no LVO (imaging misread by the PSC radiologist) (4/169), and posterior circulation occlusions (12/169). Therefore, we included 149 patients in the analysis (online supplemental figure 1). CTA was repeated in 108/149 (72%) patients and NCCT in 111/149 (74%) patients. No hemorrhages were reported in the repeated CT imaging.

10.1136/neurintsurg-2021-017441.supp1 Supplementary data

Early recanalization occurred in 48/149 (32%) patients. Spontaneous early recanalization (without IVT) occurred in 6/48 (12%) ER-LVO patients. Among the NER-LVO patients, 48/101 (48%) patients had a persistent LVO in the same vessel segment, 11/101 (11%) patients had a distally migrated thrombus, and 1/101 (1%) patients had a thrombus in a new vascular territory. For 41/101 (40%) patients, the location of the persistent LVO was not explicitly reported. Among the NER-LVO patients, we did not identify any complete thrombus resolution on the first digital subtraction angiography (DSA) scan. In four NER-LVO patients, the location of the thrombus (as shown in the DSA scan) was too distal for EVT.

ER-LVO patients less often had a medical history of hypertension than NER-LVO patients (31% vs 49%, P=0.04). NIHSS_CSC_ scores were lower for ER-LVO patients (4 vs 14, P<0.01), and median ΔNIHSS score was −5 for ER-LVO patients (clinical improvement) and 3 for NER-LVO patients (clinical deterioration) (P<0.01) (table 1). IVT was more common in ER-LVO than in NER-LVO patients (88% vs 78%), but this difference was not statistically significant (P=0.18).

**Table 1:** Baseline characteristics for ER-LVO and NER-LVO patients.

Baseline clinical characteristics*	ER-LVO, n=48	NER-LVO, n=101	P-value
Age – median (IQR)	74 (59–84)	76 (63–84)	0.78
Sex, female – no./ total (%)	27/48 (56%)	55/101 (54%)	0.84
Medical history – no./total (%)			
Previous stroke	8/48 (17%)	18/100 (18%)	0.84
Diabetes mellitus	7/48 (15%)	11/100 (11%)	0.53
Coronary artery disease	1/48 (2%)	9/100 (9%)	0.17
Hypertension	**15/48** (**31%**)	**49/100** (**49%**)	**0.04**
Atrial fibrillation	9/48 (19%)	24/100 (24%)	0.47
Pre-stroke mRS – no./total (%)			0.72
0	7/19 (37%)	10/37 (27%)	
1	11/19 (58%)	25/37 (67%)	
2	0/19 (0%)	1/37 (3%)	
3	1/19 (5%)	1/37 (3%)	
Systolic blood pressure^†^ (mmHg) – median (IQR)	147 (134–163)	150 (126–168)	0.93
Diastolic blood pressure^†^ (mmHg) – median (IQR)	83 (72–92)	82 (71–90)	0.90
Pulse pressure^†^ (mmHg) median (IQR)	68 (50–80)	63 (50–80)	0.95
NIHSS_PSC_^‡^ – median (IQR)	10 (7–15)	11 (7–16)	0.75
NIHSS_CSC_^§^ – median (IQR)	**4 (1–9**)	**14 (7–19**)	**<0.01**
ΔNIHSS^‡^ – median (IQR)	−**5 (−3-(−9**))	**3 (7-(−3**))	**<0.01**
IVT – no./total (%)	42/48 (88%)	79/101 (78%)	0.18

Number of missing values: ^†^5, ^‡^9, ^§^2.

*All baseline characteristics were measured on arrival at the CSC, unless reported otherwise.

CSC, comprehensive stroke center; ER-LVO, early-recanalized large vessel occlusion; IVT, intravenous treatment with alteplase; mRS, modified Rankin Scale; NER-LVO, non-early-recanalized large vessel occlusion; NIHSS, National Institutes of Health Stroke Scale; ΔNIHSS, change in NIHSS score between presentation to the PSC and presentation to the CSC; NIHSS_CSC_, NIHSS on CSC admission; NIHSS_PSC_, NIHSS on PSC admission; PSC, primary stroke center.

A subgroup analysis comparing the baseline characteristics of ER-LVO and NER-LVO patients that received IVT can be found in the online supplemental table 1. This analysis showed similar results to table 1: ER-LVO patients treated with IVT had lower NIHSS_CSC_ scores and more often had clinical improvement between PSC and CSC admission.

### Thrombus imaging characteristics

NCCT and CTA acquired prior to IVT were both available in 133/149 patients. Scan co-registration succeeded in 100/133 patients: 71/100 with rigid-registration and 29/100 with affine-registration. Co-registration of the remaining patients was not successful due to poor quality scans (13/33), and other co-registration errors (20/33). In total, 63 NCCT scans were re-sliced.

ER-LVO patients did not have ICA occlusions (0% vs 27%, P<0.01) and more often had M2 occlusions (35% vs 17%, P=0.01) (table 2). ER-LVO patients had longer DT (27 mm vs 12 mm, P<0.01) and shorter thrombi (17 mm vs 27 mm, P<0.01). CBS was higher in ER-LVO patients (8 vs 6, P<0.01).

**Table 2:** Thrombus imaging characteristics for ER-LVO and NER-LVO patients at baseline CT imaging.

Thrombus imaging characteristics	ER-LVO, n=48	NER-LVO, n=101	P-value
Occlusion location – no./total (%)			
ICA	**0/48** (**0%**)	**27/101** (**27%**)	**<0.01**
M1	31/48 (65%)	57/101 (56%)	0.35
M2	**17/48** (**35%**)	**17/101** (**17%**)	**0.01**
DT (mm) – median (IQR)	**27 (14–33**)	**12 (0–23**)	**<0.01**
Thrombus length (mm) – median (IQR)	**17 (10–21**)	**27 (15–38**)	**<0.01**
Thrombus perviousness (HU) – median (IQR)	13.6 (7.1–22.6)	11.0 (1.4–21.1)	0.29
Thrombus attenuation (HU) – median (IQR)	42.7 (36.8–47.4)	46.0 (36.5–55.0)	0.16
CBS – median (IQR)	**8 (7–9**)	**6 (5–8**)	**<0.01**

Occlusion location was assessed in all 149 patients. DT was measured in 131/149 patients, thrombus length in 117/149, thrombus density and perviousness in 73/149, and CBS in 124/149.

CBS, clot burden score; DT, distance from terminus of intracranial carotid artery to the thrombus; ER-LVO, early-recanalized large vessel occlusion; ICA, internal carotid artery; M1, middle cerebral artery M1 segment; M2, middle cerebral artery M2 segment; NER-LVO, non-early-recanalized large vessel occlusion.

Among patients that had IVT administered, ER-LVO patients had more distal occlusions, shorter thrombi, and higher clot burden score than NER-LVO patients. This subgroup analysis can be found in the online supplemental table 2.

### Workflow time metrics

There were no significant differences in the time metrics between ER-LVO and NER-LVO patients (online supplemental table 3).

### Outcomes

**Figure 2:**
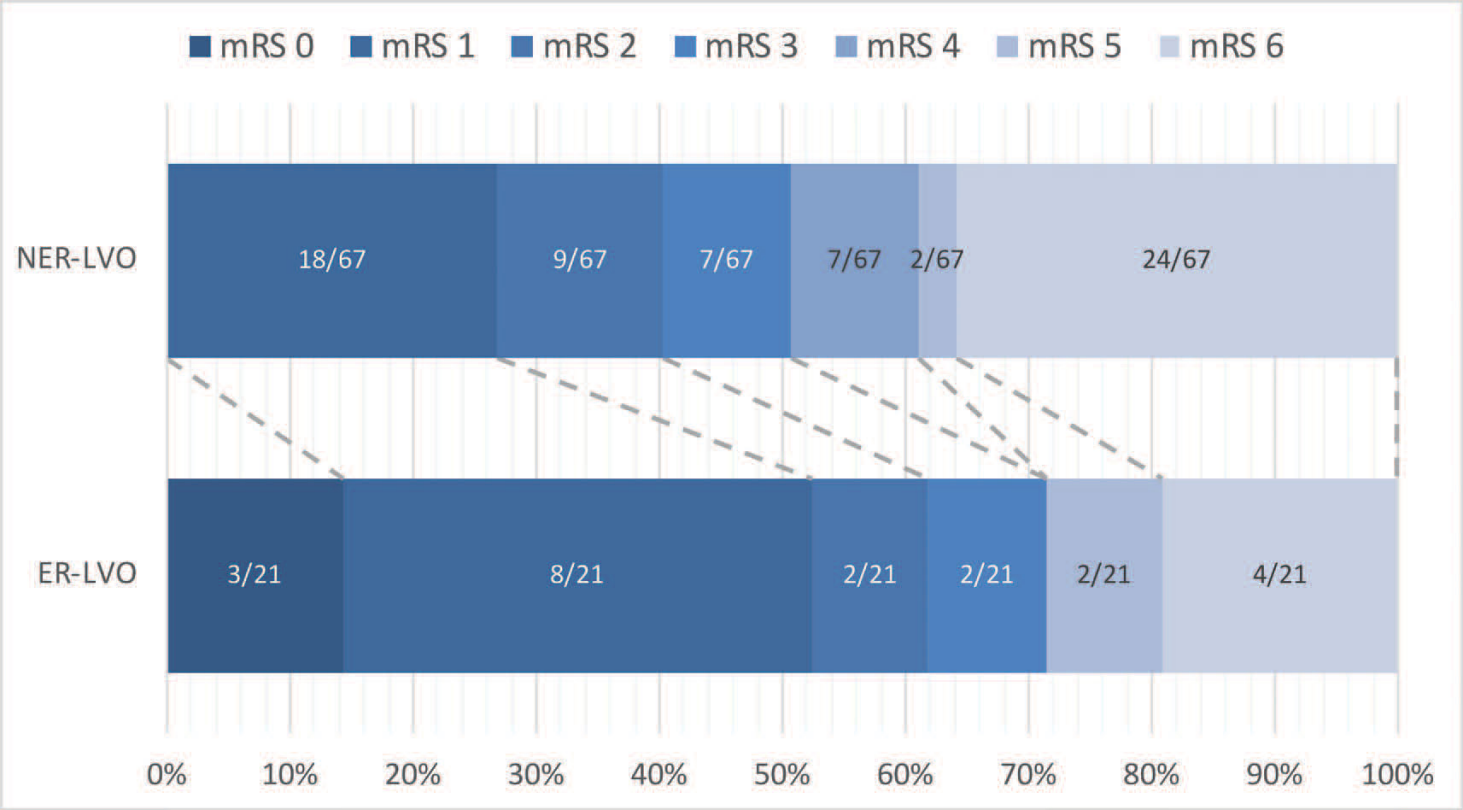
Functional outcomes according to the mRS at 90 days for ER-LVO and NER-LVO patients. ER-LVO, early-recanalized large-vessel occlusion; mRS, modified Rankin Scale; NER-LVO, non-early-recanalized large vessel occlusion.

In ER-LVO patients, mRS at 90 days was lower (1 vs 3, P=0.02) (figure 2). ER-LVO patients were more often functionally independent (62% vs 40%), however, this difference is not statistically significant (P=0.08). There were no significant differences in mortality (19% vs 36%, P=0.19) or in the occurrence of symptomatic intracranial hemorrhage (0/48 [0%] vs 2/100 [2%], P=0.99).

## Discussion

Early recanalization occurred in 32% of repeated imaging patients transferred to a CSC. These patients had more distal occlusions and shorter thrombi at baseline CT imaging. Spontaneous early recanalization occurred in a small subgroup of patients that did not receive IVT. Patients with early recanalization more often had clinical improvement between PSC and CSC admission. Early recanalization was associated with better functional outcome.

Our results are in accordance with previous findings. A systematic review of 26 studies has reported that the incidence of early recanalization after IVT is 33% (95% CI 27 to 40). Distal LVOs have been associated with less severe neurological deficit,^15^ and have more often shown early recanalization after IVT than patients with more proximal LVOs.^5 16–20^ Other studies have reported that early recanalization more often occurs in patients with shorter thrombi.^5 21–23^ In addition, shorter thrombus length has been related to distal occlusions.^13^ Previous research has reported that ER-LVO patients have better functional outcome.^17 24^ The less time the vessel is occluded, the less brain damage (time is brain), which might be translated into better patient outcome.^25^

Other studies have shown that ER-LVO patients have lower NIHSS_PSC_ scores.^4 5 16^ However, we only found statistically significant differences in the NIHSS_CSC_ and the ΔNIHSS scores, which likely reflect the clinical improvement of the patient due to the thrombus resolution. Previous studies have also suggested that higher thrombus attenuation and partial occlusions are potential predictors of early recanalization.^5 21 26 27^ However, we did not find any differences in these characteristics between ER-LVO and NER-LVO patients.

We found that patients with a medical history of hypertension were less likely to have early recanalization. High blood pressure on hospital admission has been related to reducing the chances of early recanalization.^28^ However, we did not find any differences in the systolic, diastolic, or pulse pressure on CSC admission.

Early recanalization is a factor to consider in the current debate on which treatment is more beneficial for AIS patients: bridging therapy (IVT together with EVT) or EVT alone. Some previous studies found that patients with bridging therapy have a better functional outcome,^29^ while others have suggested that bridging therapy does not improve functional outcome over EVT alone.^30^ Our study suggests that: the administration of IVT in patients that are far from a CSC is important and may lead to a better functional outcome of these patients; and repeated imaging in the CSC might reduce futile transfers to the angio-suite for EVT (and thus, invasive angiographic imaging and associated procedural risks^9^) and improve efficient use of resources. Therefore, early recanalization might be relevant when designing the treatment workflow of patients who are considered for transfer to a CSC. Future research might focus on developing a prediction model of early recanalization in order to improve the workflow of patients considered for transfer to a CSC for EVT (eg, by identifying beforehand patients who do not benefit from IVT).

### Limitations

Our study has some limitations. First, the number of transferred patients who underwent repeated imaging is limited, and only one-third shows early recanalization. Therefore, any statistical analysis comparing both groups is also limited. In addition, 41% of mRS scores at 90 days' post-stroke were missing.

Second, the requirements for measuring the thrombus imaging characteristics further reduced our sample size. The slice thickness exceeded 2.5 mm in 63 NCCT scans. The HU interpolation caused by the re-slicing led to the exclusion of several patients for the thrombus density and perviousness measurements. Moreover, defining the thrombus extension was challenging when both modalities were not available, especially in cases without collateral filling on CTA and in the absence of the hyperdense artery sign on NCCT, which in many cases prevent us from measuring thrombus length. In addition, the performed measurements are observer-dependent. An (semi-) automatized method that places the markers along the occluded vessel centerline would be less time-consuming and would reduce the observer-dependency.

Finally, the decision to perform repeated imaging is not arbitrary.^3^ ER-LVO patients might be more likely to undergo repeated imaging than NER-LVO patients.

## Conclusion

Patients with early recanalization are characterized by more distal occlusions and shorter thrombi at baseline CT imaging, and clinical improvement between presentation to the PSC and the CSC. Early recanalization is associated with better functional patient outcome and might therefore be an important factor to consider in treatment-planning of transferred stroke patients.

## Data Availability

All data relevant to the study are included in the article or uploaded as supplementary information. The data that support the findings of this study are available upon reasonable request, after clearance by the local ethics committee.

## References

[1] Powers WJ , Derdeyn CP , Biller J , et al . 2015 American Heart Association/American Stroke Association focused update of the 2013 guidelines for the early management of patients with acute ischemic stroke regarding endovascular treatment. Stroke 2015;46:3020–35. 10.1161/STR.0000000000000074 26123479

[2] Fuentes B , Alonso de Leciñana M , Ximénez-Carrillo A , et al . Futile interhospital transfer for endovascular treatment in acute ischemic stroke: the Madrid Stroke Network experience. Stroke 2015;46:2156–61. 10.1161/STROKEAHA.115.009282 26106117

[3] van Meenen LCC , Arrarte Terreros N , Groot AE . Value of repeated imaging in patients with a stroke who are transferred for endovascular treatment. J Neurointerv Surg 2021;0:1–5. 10.1136/neurintsurg-2020-017050 33685983 PMC8784993

[4] Seners P , Turc G , Naggara O , et al . Post-thrombolysis recanalization in stroke referrals for thrombectomy: incidence, predictors, and prediction scores. Stroke 2018;49:2975–82. 10.1161/STROKEAHA.118.022335 30730694

[5] Seners P , Turc G , Maïer B , et al . Incidence and predictors of early recanalization after intravenous thrombolysis: a systematic review and meta-analysis. Stroke 2016;47:2409–12. 10.1161/STROKEAHA.116.014181 27462117

[6] Sablot D , Dumitrana A , Leibinger F , et al . Futile inter-hospital transfer for mechanical thrombectomy in a semi-rural context: analysis of a 6-year prospective registry. J Neurointerv Surg 2019;11:539–44. 10.1136/neurintsurg-2018-014206 30327386

[7] Miller DJ , Simpson JR , Silver B . Safety of thrombolysis in acute ischemic stroke: a review of complications, risk factors, and newer technologies. Neurohospitalist 2011;1:138–47. 10.1177/1941875211408731 23983849 PMC3726129

[8] Ohara T , Menon BK , Al-Ajlan FS , et al . Thrombus migration and fragmentation after intravenous alteplase treatment: the INTERRSeCT study. Stroke 2021;52:203–12. 10.1161/STROKEAHA.120.029292 33317416

[9] Khatri R , Maud A , Rodriguez GJ . Complications During Mechanical Thrombectomy: Pitfalls and Bailouts. In: Samaniego E , Hasan D , eds. Acute stroke management in the era of thrombectomy. Cham: Springer, 2019: 173–90.

[10] Klein S , Staring M , Murphy K . Elastix : a toolbox for intensity-based medical image registration. IEEE Transactions on Medical Imaging 2010;29:196–205. 10.1109/TMI.2009.2035616 19923044

[11] Muschelli J . Recommendations for processing head CT data. Front Neuroinform 2019;13:1–9. 10.3389/fninf.2019.00061 31551745 PMC6738271

[12] Yushkevich PA , Piven J , Hazlett HC , et al . User-guided 3D active contour segmentation of anatomical structures: significantly improved efficiency and reliability. Neuroimage 2006;31:1116–28. 10.1016/j.neuroimage.2006.01.015 16545965

[13] Dutra BG , Tolhuisen ML , Alves HCBR , et al . Thrombus imaging characteristics and outcomes in acute ischemic stroke patients undergoing endovascular treatment. Stroke 2019;50:2057–64. 10.1161/STROKEAHA.118.024247 31216961

[14] Tan IYL , Demchuk AM , Hopyan J , et al . CT angiography clot burden score and collateral score: correlation with clinical and radiologic outcomes in acute middle cerebral artery infarct. AJNR Am J Neuroradiol 2009;30:525–31. 10.3174/ajnr.A1408 19147716 PMC7051470

[15] Tian H , Parsons MW , Levi CR , et al . Influence of occlusion site and baseline ischemic core on outcome in patients with ischemic stroke. Neurology 2019;92:e2626–43. 10.1212/WNL.0000000000007553 31043475

[16] Murphy A , Symons SP , Hopyan J , et al . Factors influencing clinically meaningful recanalization after IV-rtPA in acute ischemic stroke. AJNR 2013;34:146–52. 10.3174/ajnr.A3169 22700751 PMC7966325

[17] Zangerle A , Kiechl S , Spiegel M , et al . Recanalization after thrombolysis in stroke patients: predictors and prognostic implications. Neurology 2007;68:39–44. 10.1212/01.wnl.0000250341.38014.d2 17200490

[18] Lee K-Y , Han SW , Kim SH , et al . Early recanalization after intravenous administration of recombinant tissue plasminogen activator as assessed by pre- and post-thrombolytic angiography in acute ischemic stroke patients. Stroke 2007;38:192–3. 10.1161/01.STR.0000251788.03914.00 17110611

[19] Mueller L , Pult F , Meisterernst J , et al . Impact of intravenous thrombolysis on recanalization rates in patients with stroke treated with bridging therapy. Eur J Neurol 2017;24:1016–21. 10.1111/ene.13330 28649759

[20] Demchuk AM , Goyal M , Yeatts SD , et al . Recanalization and clinical outcome of occlusion sites at baseline CT angiography in the interventional management of stroke III trial. Radiology 2014;273:202–10. 10.1148/radiol.14132649 24895878 PMC4174723

[21] Mishra SM , Dykeman J , Sajobi TT , et al . Early reperfusion rates with IV tPA are determined by CTA clot characteristics. AJNR 2014;35:2265–72. 10.3174/ajnr.A4048 25059699 PMC7965306

[22] Behrens L , Möhlenbruch M , Stampfl S , et al . Effect of thrombus size on recanalization by bridging intravenous thrombolysis. Eur J Neurol 2014;21:1406–10. 10.1111/ene.12509 25040586

[23] Seners P , Delepierre J , Turc G , et al . Thrombus length predicts lack of post-thrombolysis early recanalization in minor stroke with large vessel occlusion. Stroke 2019;50:761–4. 10.1161/STROKEAHA.118.023455 30802186

[24] Ospel JM , Singh N , Almekhlafi MA , et al . Early recanalization with alteplase in stroke because of large vessel occlusion in the ESCAPE trial. Stroke 2021;52:304–7. 10.1161/STROKEAHA.120.031591 33213288

[25] Saver JL . Time is brain--quantified. Stroke 2006;37:263–6. 10.1161/01.STR.0000196957.55928.ab 16339467

[26] Ahn SH , d'Esterre CD , Qazi EM , et al . Occult anterograde flow is an under-recognized but crucial predictor of early recanalization with intravenous tissue-type plasminogen activator. Stroke 2015;46:968–75. 10.1161/STROKEAHA.114.008648 25700286

[27] Puig J , Pedraza S , Demchuk A , et al . Quantification of thrombus Hounsfield units on noncontrast CT predicts stroke subtype and early recanalization after intravenous recombinant tissue plasminogen activator. AJNR 2012;33:90–6. 10.3174/ajnr.A2878 22158924 PMC7966152

[28] Mattle HP , Kappeler L , Arnold M , et al . Blood pressure and vessel recanalization in the first hours after ischemic stroke. Stroke 2005;36:264–8. 10.1161/01.STR.0000153052.59113.89 15637309

[29] Mistry EA , Mistry AM , Nakawah MO , et al . Mechanical thrombectomy outcomes with and without intravenous thrombolysis in stroke patients: a meta-analysis. Stroke 2017;48:2450–6. 10.1161/STROKEAHA.117.017320 28747462

[30] Coutinho JM , Liebeskind DS , Slater L-A , et al . Combined intravenous thrombolysis and thrombectomy vs thrombectomy alone for acute ischemic stroke: a pooled analysis of the SWIFT and STAR studies. JAMA Neurol 2017;74:268–74. 10.1001/jamaneurol.2016.5374 28097310

