# Supplementary material for: Early recanalization in large-vessel occlusion stroke patients transferred for endovascular treatment
Source: J Neurointerv Surg. 2021 May 13;14(5):480–4. doi: 10.1136/neurintsurg-2021-017441 (PMC9016237; doi:10.1136/neurintsurg-2021-017441)
Supplement: Supplementary data [file neurintsurg-2021-017441supp001.pdf]

SUPPLEMENTAL MATERIAL

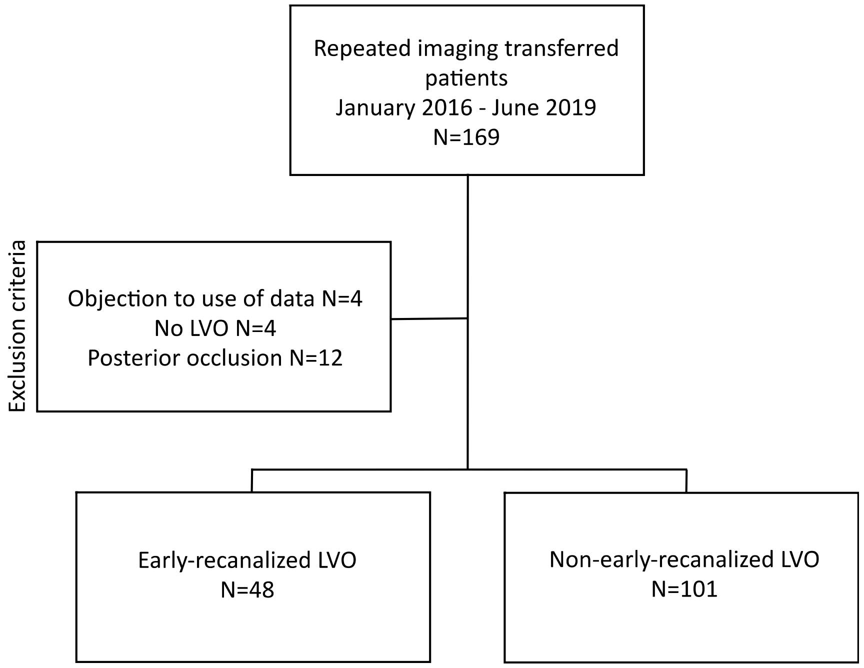

Supplemental Figure 1: Inclusion flow chart. The abbreviation stands for: LVO, large vessel occlusion.

Arrarte Terreros N, *et al. J NeuroIntervent Surg* 2022; 14:480–484. doi: 10.1136/neurintsurg-2021-017441

Supplemental Table 1: Baseline characteristics for ER-LVO and NER-LVO patients that received intravenous treatment with alteplase (IVT).

Baseline clinical characteristics* - IVT subgroup	ER-LVO, n=42	NER-LVO, n=79	p-value
Age – median (IQR)	74 (59-84)	72 (59-84)	0.92
Sex, female – no./ total (%)	23/42 (55%)	42/79 (53%)	0.87
Medical history – no./total (%)			
Previous stroke	6/42 (14%)	12/79 (15%)	0.89
Diabetes mellitus	7/42 (17%)	10/79 (13%)	0.55
Coronary artery disease	1/42 (2%)	6/79 (8%)	0.24
Hypertension	12/42 (29%)	37/79 (47%)	0.05
Atrial fibrillation	7/42 (17%)	14/79 (18%)	0.88
Pre-stroke mRS – no./total (%)			0.75
0	6/16 (38%)	9/32 (28%)	
1	9/16 (56%)	21/32 (66%)	
2	0/16 (0%)	1/32 (3%)	
3	1 /16 (6%)	1/32 (3%)	
Systolic blood pressure§ [mmHg] – median (IQR)	143 (130-163)	148 (125-166)	0.98
Diastolic blood pressure§ [mmHg] – median (IQR)	80 (70-90)	83 (71-90)	0.56
Pulse pressure§ [mmHg] median (IQR)	69 (49-80)	62 (48-79)	0.70
$NIHSS_{PSC}$ † – median (IQR)	10 (7-15)	12 (8-17)	0.36
$NIHSS_{CSC}$ ‡ – median (IQR)	**4 (1-9)**	**14 (7-20)**	**<0.01**
ΔNIHSS† – median (IQR)	**-5 (-3-(-8))**	**3 (7-(-2))**	**<0.01**

CSC, comprehensive stroke center; ΔNIHSS, change in NIHSS score between presentation to the PSC and presentation to the CSC; ER-LVO, early-recanalized large vessel occlusion; IVT, intravenous treatment with alteplase; mRS, modified Rankin scale; NER-LVO, non-early-recanalized large vessel occlusion; NIHSS, National Institutes of Health Stroke Scale; $NIHSS_{CSC}$, NIHSS on CSC admission; $NIHSS_{PSC}$, NIHSS on PSC admission; PSC, primary stroke center.

*All baseline characteristics were measured on arrival at the CSC, unless reported otherwise.

Number of missing values: †7, ‡2, §4.

Arrarte Terreros N, *et al. J NeuroIntervent Surg* 2022; 14:480–484. doi: 10.1136/neurintsurg-2021-017441

Supplemental Table 2: Thrombus imaging characteristics at baseline CT imaging for ER-LVO and NER-LVO patients that received intravenous treatment with alteplase (IVT).

**Thrombus imaging characteristics – IVT subgroup**	**ER-LVO, n=42**	**NER-LVO, n=79**	**p-value**
Occlusion location – no./total (%)			
ICA	**0/42 (0%)**	**22/79 (28%)**	**<0.01**
M1	27/42 (64%)	44/79 (56%)	0.36
M2	**15/42 (36%)**	**13/79 (16%)**	**0.02**
DT [mm] – median (IQR)	**27 (15-33)**	**10 (0-22)**	**<0.01**
Thrombus length [mm] – median (IQR)	**16 (10-19)**	**28 (16-48)**	**<0.01**
Thrombus perviousness [HU] – median (IQR)	13.5 (7.7-27.8)	12.3 (0.7-22.0)	0.30
Thrombus attenuation [HU] – median (IQR)	42.8 (37.8-47.6)	46.4 (36.2-56.1)	0.35
CBS – median (IQR)	**8 (7-9)**	**6 (5-8)**	**<0.01**

CBS, clot burden score; DT, distance from terminus of intracranial carotid artery to the thrombus; ER-LVO, early-recanalized large vessel occlusion; ICA, internal carotid artery; M1, middle cerebral artery M1 segment; M2, middle cerebral artery M2 segment; NER-LVO, non-early-recanalized large vessel occlusion.

Occlusion location was assessed in all 121 patients. DT was measured in 107/121 patients, thrombus length in 95/121, thrombus density and perviousness in 58/121, and CBS in 101/121.

Supplemental Table 3: Workflow-related time metrics for ER-LVO and NER-LVO patients.

Workflow-related time metrics	ER-LVO, n=48	NER-LVO, n=101	p-value
Onset to door time* [min] – median (IQR)	48 (37-118)	55 (38-90)	0.91
Onset to needle time† [min] – median (IQR)	65 (47-71)	83 (58-116)	0.35
Door to needle time‡ [min] – median (IQR)	20 (20-21)	23 (18-30)	0.44
PSC imaging to CSC imaging time§ [min] – median (IQR)	90 (74-114)	93 (72-124)	0.25

CSC, comprehensive stroke center; Door to needle time, time from arrival at the PSC to IVT initiation; ER-LVO, early-recanalized large vessel occlusion; NER-LVO, non-early-recanalized large vessel occlusion; Onset to door time, time from stroke onset to arrival at the PSC; Onset to needle time, time from stroke onset to IVT initiation; PSC, primary stroke center; PSC imaging to CSC imaging time, time difference between the imaging taken at the PSC and the repeated imaging performed at the CSC.

Number of missing values: *83, †96, ‡109, §19.

Arrarte Terreros N, *et al. J NeuroIntervent Surg* 2022; 14:480–484. doi: 10.1136/neurintsurg-2021-017441
